# Former Very Preterm Infants Show Alterations in Thyroid Function at a Preschool Age

**DOI:** 10.1155/2017/3805370

**Published:** 2017-07-19

**Authors:** Anna Posod, Irena Odri Komazec, Ulrike Pupp Peglow, Dagmar Meraner, Elke Griesmaier, Ursula Kiechl-Kohlendorfer

**Affiliations:** ^1^Pediatrics II (Neonatology), Department of Pediatrics, Medical University of Innsbruck, Anichstrasse 35, 6020 Innsbruck, Austria; ^2^Pediatrics III (Pediatric Cardiology), Department of Pediatrics, Medical University of Innsbruck, Anichstrasse 35, 6020 Innsbruck, Austria; ^3^Pediatrics I (Pediatric Endocrinology), Department of Pediatrics, Medical University of Innsbruck, Anichstrasse 35, 6020 Innsbruck, Austria

## Abstract

Preterm birth is frequently associated with altered thyroid hormone levels in the newborn period. Recent data suggest a role of prematurity independent of birth size also in childhood thyroid dysfunction. Whether the high-risk population of former very preterm infants (VPI) is particularly susceptible to thyroid hormone alterations is currently unknown. The aim of the present study was to assess whether former VPI display changes in thyroid hormone status in comparison to term-born controls at a preschool age. Free triiodothyronine (fT3), free thyroxine (fT4), and thyroid stimulating hormone (TSH) concentrations were determined in former VPI and same-aged children born at term at five to seven years of age. 31 former term infants and 82 former VPI were included in the study. In comparison to children born at term, former VPI had lower fT4 (16.1 ± 1.8 versus 17.0 ± 2.1 pmol/l), higher fT3 (6.8 ± 0.7 versus 6.5 pmol/l), and higher TSH levels (3.0 ± 1.4 versus 2.3 ± 1.0 *μ*U/l), independent of major neonatal morbidities. As subclinical changes in thyroid hormone status are potentially associated with adverse health profiles, close follow-up of these children is warranted.

## 1. Introduction

Preterm birth is frequently associated with altered thyroid hormone levels in the newborn period, especially in the presence of major morbidities [1–4]. Recent data suggest a role of prematurity also in childhood thyroid dysfunction [4, 5]. Whether the especially vulnerable population of very preterm infants (VPI) born at less than 32 weeks' gestation is at particular risk of long-lasting thyroid dysfunction is unknown to date. The aim of the present study was to determine thyroid hormone levels in former VPI at a preschool age. We hypothesized that thyroid hormone alterations are detectable in former VPI in comparison to same-aged children born at term.

## 2. Materials and Methods

The current investigation is part of the cross-sectional study “Preterm infants and early markers for an increased risk of cardiovascular disease,” which examined former VPI and term-born controls at five to seven years of age at Innsbruck University Hospital, Austria, from May 2012 to March 2015. All participants undergoing fasting blood sampling at study visit were considered eligible for thyroid hormone measurements. The detailed study protocol has been published elsewhere [6–8]. The following additions were made for thyroid function assessment: With regard to perinatal characteristics in the VPI cohort, respiratory support was graded by invasiveness of measure taken; categorization followed the most invasive mode applied in each subject. Severe infection/inflammation was defined as a diagnosis of early onset sepsis and/or late onset sepsis and/or necrotizing enterocolitis in the newborn period. At study visit, free triiodothyronine (fT3), free thyroxine (fT4), and thyroid stimulating hormone (TSH) concentrations were determined in undiluted serum samples using the Advia® Centaur™ Immunoassay System (Siemens Healthcare Diagnostics, Vienna, Austria) as described previously [9]. Abnormally high fT3, fT4, and TSH concentrations were defined as values above the 97.5th percentile (P97.5) and abnormally low concentrations as values below the 2.5th percentile (P2.5) according to age- and gender-specific pediatric reference intervals [9].

Statistical analyses were carried out with SPSS for Windows, version 24 (SPSS Inc., Armonk, NY, USA). Representativeness of samples was assured by comparison with reference populations (term: SIDS database Tyrol, birth years 2007–2009; preterm: Innsbruck routine VPI follow-up database, birth years 2007–2009).

Differences in perinatal characteristics and characteristics at study visit were determined with Fisher's Exact Test or Mann–Whitney *U* Test. Differences in thyroid hormone levels were assessed by means of logistic regression analysis. Input data were logarithmically transformed prior to analyses. Covariate adjustments were made stepwise: Model A was adjusted for age at examination and sex, model B for all parameters of model A plus birth weight *z*-score and current body mass index (BMI) *z*-score, and model C for all parameters of model B plus maternal educational status and smoking during pregnancy. Odds ratios and respective confidence intervals were calculated as odds ratios per standard deviation (SD) increase. Subgroup analyses were conducted with Mann–Whitney *U* Test or Kruskal-Wallis Test and Bonferroni correction for multiple comparisons.

All followed procedures were in accordance with the ethical standards of the responsible committee on human experimentation and with the Helsinki Declaration of 1975, as revised in 1983. Ethical approval was obtained from the institutional review board of the Medical University of Innsbruck. Written informed consent was obtained from all legal guardians and verbal consent from all study participants prior to inclusion in the study.

## 3. Results

34 children born at term and 84 children born very preterm were eligible for the study. Thyroid hormone measurements were available in 31 former term infants and 82 former VPI. Both study groups did not differ from respective reference populations in regard to sex distribution, birth weight, or maternal educational status. Gestational age in the preterm study group was similar to all VPI in the survey area. Current BMI and current BMI *z*-score were significantly lower in former VPI in comparison to term-born controls (Table 1). All subjects underwent dried blood spot screening for congenital hypothyroidism in the newborn period as part of the National Austrian Newborn Screening Program for inherited metabolic and endocrinologic disorders (routine sampling at 36–72 hours of life in all subjects, additional sampling in preterm infants at ≥32 weeks postmenstrual age) [10–12]. Neonatal TSH values were within a normal range for the age group (<15 *μ*U/ml) in all study participants; no recalls were required (personal communication National Laboratory for Newborn Screening, Medical University of Vienna, Austria). None of the study participants were known to have been exposed to topical iodine. In the VPI group, amidotrizoic acid-containing contrast agents (Gastrografin®, iodine content 367 mg/ml) were used in three cases to facilitate meconium passage in the neonatal period. Five former VPI received thyroxine treatment in a dosage of approximately 100 *μ*g/m^2^ surface area per day due to abnormal thyroid hormone concentrations and clinical signs of hypothyroidism (median hormone concentration at treatment initiation (interquartile range, IQR): fT3: 4.1 (2.7, 4.4) pmol/l; fT4: 14.9 (10.6, 21.5) pmol/l; TSH: 5.3 (3.5, 5.6) *μ*U/ml; median age at treatment initiation (IQR): 10.5 (3.5, 25.0) days; median duration of treatment (IQR): 50.5 (27, 76) days; median hormone concentration at treatment discontinuation (IQR): fT3: 4.9 (4.4, 6.2) pmol/l; fT4: 17.4 (16.2, 20.9) pmol/l; TSH: 1.0 (0.4, 4.5) *μ*U/ml). 64 VPI received a full course of antenatal glucocorticoids; seven received an incomplete course. No glucocorticoids were given in eight cases; in three cases administration was not documented. 13 VPI received glucocorticoids postnatally for treatment of bronchopulmonary dysplasia. Two VPI received no respiratory support and 39 VPI nasal continuous positive airway pressure; 30 VPI were conventionally ventilated and 11 VPI underwent high-frequency oscillation ventilation. Severe infection/inflammation was present in 30 VPI (culture-proven sepsis in 7 cases) and patent ductus arteriosus in 26 VPI. With regard to multiple births, all children born at term were singletons; in the VPI group 22 children (26.8%) were twins and 3 children (3.7%) were triplets. None of the participants were taking medication with a potential impact on thyroid function at preschool age. At study visit, fT3 levels were higher in former VPI; significance was attained after model A covariate adjustment. fT4 levels were lower in former VPI; significance was lost after model C covariate adjustment. TSH levels were significantly higher in former VPI in all models (Tables 1 and 2). Due to the low number of small for gestational age (term: *n* = 2; preterm: *n* = 9) and large for gestational age (term: *n* = 1; preterm: *n* = 4) newborns in both study groups, the low prevalence of multiple births, and the infrequent use of amidotrizoic acid-containing contrast agents as well as thyroxine replacement therapy in VPI, subgroup analyses were not undertaken. Separate analyses excluding these subjects yielded similar results with comparable effect sizes in this regard. In the term study group, abnormally high fT3 concentrations were detected in two cases, abnormally high fT4 concentrations in one case, and abnormally low TSH concentrations in another case. In former VPI, abnormally high fT3 concentrations were detected in six cases and abnormally high TSH concentrations in three cases. Prevalence did not significantly differ between groups (fT3 > P97.5: *p* = 1.000, fT3 < P2.5: no cases in either group; fT4 > P97.5: *p* = 0.274, fT4 < P2.5: no cases in either group; TSH > P97.5: *p* = 0.558, TSH < P2.5: *p* = 0.279; Fisher's Exact Test). In former VPI, thyroid hormone levels were independent of use of ante- (fT3: *p* = 0.114, fT4: *p* = 0.369, and TSH: *p* = 0.133; Mann–Whitney *U* Test with Bonferroni correction, adjusted significance level *α* = 0.007) or postnatal systemic glucocorticoids (fT3: *p* = 0.894, fT4: *p* = 0.703, and TSH: *p* = 0.744; Mann–Whitney *U* Test with Bonferroni correction, adjusted significance level *α* = 0.007), respiratory support modes (fT3: *p* = 0.739, fT4: *p* = 0.319, and TSH: *p* = 0.455; Kruskal-Wallis Test with Bonferroni correction, adjusted significance level *α* = 0.007), presence of severe infection/inflammation (fT3: *p* = 0.312, fT4: *p* = 0.567, and TSH: *p* = 0.365; Mann–Whitney *U* Test with Bonferroni correction, adjusted significance level *α* = 0.007), or patent ductus arteriosus (fT3: *p* = 0.540, fT4: *p* = 0.300, and TSH: *p* = 0.696; Mann–Whitney *U* Test with Bonferroni correction, adjusted significance level *α* = 0.007) in the newborn period.

In the former VPI group, none of the children had a positive change in weight *z*-score from birth to discharge at approximately term-equivalent age (median postmenstrual age at discharge (IQR): 37 + 3 (36 + 4, 39 + 1) weeks). From discharge to first follow-up (median corrected age (IQR): 3 (3, 4) months), 38 former VPI (49.4%) had a positive change in weight *z*-score reflecting catch-up growth. fT3 and fT4 concentrations did not significantly differ between children who did or did not show catch-up growth (mean hormone concentration (SD); fT3: 6.9 (0.7) versus 6.7 (0.7) pmol/l, *p* = 0.277; fT4: 16.0 (2.1) versus 16.3 (1.5) pmol/l, *p* = 0.628; Mann–Whitney *U* Test with Bonferroni correction, adjusted significance level *α* = 0.007). TSH levels were significantly lower in children displaying catch-up growth (mean hormone concentration (SD); TSH: 2.6 (1.1) versus 3.5 (1.6) *μ*U/ml, *p* = 0.005; Mann–Whitney *U* Test with Bonferroni correction, adjusted significance level *α* = 0.007).

## 4. Discussion

Preterm birth is frequently associated with altered thyroid hormone levels in the newborn period and has also been linked to childhood thyroid dysfunction [4, 5]. Whether former VPI are at particular risk of changes in thyroid hormone status has previously been unknown. Our study shows that in comparison to children born at term former VPI have lower fT4 and higher fT3 levels at a preschool age. These findings are of questionable clinical relevance, as differences observed were marginal. However, TSH levels were considerably higher in former VPI in comparison to term-born controls, independent of perinatal characteristics and major neonatal morbidities in the VPI group.

The association between prematurity and TSH concentrations at preschool age in our study is in contrast to a population-based cohort analysis by Korevaar et al., which reported an inverse correlation of gestational age with newborn, but not childhood TSH concentrations [13]. This difference, however, might be explained by the fact that Korevaar et al. included only term- and near-term-born neonates, thus not representing the at-risk population of children born early preterm [13, 14].

Even though within a normal range, TSH alterations are potentially clinically relevant. Low-normal thyroid function has been shown to be associated with dyslipidemia, aortic atherosclerosis, and myocardial infarction in adult populations [15–18]. In addition, data suggest a potential favorable effect of thyroxine replacement in adults with subclinical hypothyroidism [19]. The exact implication of high-normal TSH concentrations in future cardiovascular health of former VPI remains to be elucidated. It is also currently unclear whether VPI with subclinical alterations in thyroid function might benefit from early thyroxine substitution. Pros and cons in this regard have to be weighed carefully, as recent data suggest that subclinical hypothyroidism is often a self-limiting condition in pediatric populations [20, 21]. Follow-up assessment of former VPI with regard to thyroid function, however, seems justified. Of interest, TSH levels in our study were significantly lower in former VPI who displayed catch-up growth from term-equivalent age to 3 months of corrected age in comparison to VPI who did not show an increase in weight *z*-scores. This is in accordance with a study by Cianfarani et al., who reported higher TSH concentrations in small for gestational age children with blunted catch-up growth [22]. In growth-restricted infants, altered intrauterine programming of hypothalamic-pituitary function has been proposed [23]. Whether this is also the case in preterm infants is unknown to date and has to be addressed by future studies.

A main limitation of our study is that, due to its design, thyroid hormone measurements were only undertaken at a single time point. Whether alterations preexisted at an earlier age or might worsen over time is thus currently still open to question. In order to shed light on thyroid function dynamics and on potential long-term consequences, both prospective trials starting in the neonatal period and extensive follow-up studies are required.

## 5. Conclusion

In comparison to children born at term, former VPI have lower fT4, higher fT3, and higher TSH concentrations at a preschool age. Findings are independent of major neonatal morbidities. As even subclinical alterations in thyroid hormone status are potentially associated with adverse health profiles, a paradigm change in institutional policies with an expansion of routine follow-up programs for former VPI might be warranted.

## Figures and Tables

**Table 1:** Perinatal characteristics and characteristics at study visit in former term and very preterm infants.

Characteristic	Term group (*n* = 31)	VPI group (*n* = 82)
Sex, male/female, *n* (%)	15 (48)/16 (52)	41 (50)/41 (50)
*Perinatal characteristics*		
Gestational age, median (IQR) [weeks]	40 (39; 41)	30 (28; 31)^*∗∗∗*^
Birth weight, mean (SD) [grams]	3340 (439)	1211 (409)^*∗∗∗*^
Birth weight *z*-score, mean (SD)	−0.24 (0.78)	−0.15 (0.94)
Small for gestational age at birth, *n* (%)	2 (6.9)	9 (11.0)
Large for gestational age at birth, *n* (%)	1 (3.2)	4 (4.9)
Maternal educational status,	5 (16)/15 (48)/11 (36)	0 (0)/47 (57)/35 (43)
unknown/<12 years/≥12 years, *n* (%)		
Smoking during pregnancy,	6 (19)/5 (16)/20 (65)	0 (0)/23 (28)/59 (72)
unknown/yes/no, *n* (%)		
*Characteristics at study visit*		
Age at examination, mean (SD) [years]	5.5 (0.7)	5.4 (0.3)
Current BMI, median (IQR) [kg/m^2^]	14.8 (14.0; 15.5)	14.0 (13.6; 14.9)^*∗∗*^
Current BMI *z*-score, mean (SD)	−0.38 (0.84)	−0.91 (1.31)^*∗∗*^
fT3, mean (SD) [pmol/l]	6.5 (0.7)	6.8 (0.7)^*∗*^
fT4, mean (SD) [pmol/l]	17.0 (2.1)	16.1 (1.8)^*∗*^
TSH, mean (SD) [*µ*U/ml]	2.3 (1.0)	3.0 (1.4)^*∗∗*^

BMI, body mass index (calculated as weight in kilograms divided by height in meters squared); fT3, free triiodothyronine; fT4, free thyroxine; IQR, interquartile range; SD, standard deviation; TSH, thyroid stimulating hormone; VPI, very preterm infant; ^*∗*^*p* < 0.05, term versus VPI group; ^*∗∗*^*p* < 0.01, term versus VPI group; ^*∗∗∗*^*p* < 0.001, term versus VPI group.

**Table 2:** Logistic regression analysis of thyroid hormone levels in children born very preterm in comparison to same-aged controls born at term.

Outcome variable and analysis model^a^	*β* coefficient	*p* value	OR (95% CI)^b^
*fT*3^c,d^			
Unadjusted	0.415	0.062	1.51 (0.98; 2.34)
Model A	0.479	*0.044*	1.62 (1.01; 2.58)
Model B	0.577	*0.021*	1.78 (1.09; 2.90)
Model C	0.678	*0.015*	1.97 (1.14; 3.39)
*fT*4^c,d^			
Unadjusted	−0.593	*0.014*	0.55 (0.35; 0.89)
Model A	−0.503	*0.047*	0.61 (0.37; 0.99)
Model B	−0.560	*0.033*	0.57 (0.34; 0.95)
Model C	−0.570	0.054	0.57 (0.32; 1.01)
*TSH* ^c,d^			
Unadjusted	0.680	*0.004*	1.97 (1.24; 3.14)
Model A	0.679	*0.011*	1.97 (1.17; 3.32)
Model B	0.641	*0.015*	1.90 (1.13; 3.19)
Model C	0.663	*0.019*	1.94 (1.12; 3.40)

BMI, body mass index (calculated as weight in kilograms divided by height in meters squared); CI, confidence interval; fT3, free triiodothyronine; fT4, free thyroxine; OR, odds ratio(s); TSH, thyroid stimulating hormone. ^a^Model A was adjusted for age at examination and sex. Model B was adjusted for all parameters of model A plus birth weight *z*-score and current BMI *z*-score. Model C was adjusted for all parameters of model B plus maternal educational status and smoking during pregnancy. ^b^OR were calculated for former preterm infants in relation to same-aged children born at term. ^c^OR are reported as OR per SD increase. ^d^Input data were logarithmically transformed for analyses.
